# Minimally invasive surgery for large hiatal hernia

**DOI:** 10.1002/ags3.12278

**Published:** 2019-07-17

**Authors:** Nobuo Omura, Kazuto Tsuboi, Fumiaki Yano

**Affiliations:** ^1^ Department of Surgery National Hospital Organization Nishisaitama‐Chuo National Hospital Tokyo Japan; ^2^ Department of Surgery The Jikei University School of Medicine Tokyo Japan

**Keywords:** elective repair, large hiatal hernia, mesh reinforcement, relaxing incision

## Abstract

The majority of large hiatal hernias are paraesophageal hiatal hernias (PEH). Once prolapse of the stomach to the chest cavity reaches a high degree, it is called an intrathoracic stomach. More than 25 years have elapsed since laparoscopic surgery was carried out as minimally invasive surgery for PEH. The feasibility and safety thereof has nearly been established. PEH may cause serious complications such as strangulation and perforation. The outcome of elective repair of PEH is better than emergent repair, so we should carry out elective repair as much as possible. Although not a major clinical problem, following PEH repair the rate of anatomical recurrence increases with age. In order to reduce the recurrence rate, mesh reinforcement by crural repair has been widely performed. Although this improves the short‐term outcomes, the long‐term outcomes are unclear. For PEH repair, fundoplication and gastropexy are believed desirable. We should select the procedure associated with a lower incidence of dysphagia and so on following surgery. While relaxing incision is useful for primary tension‐free closure, it has not contributed to improvement in the recurrence rate.

## 1 INTRODUCTION

Nearly 30 years have elapsed since the first report on laparoscopic surgery for gastroesophageal reflux disease (GERD) by Dallemagne et al in 1991. Although the surgery for paraesophageal hiatal hernias (PEH) is more technically difficult than for sliding esophageal hiatal hernias, Cushieri et al^1^ in 1992 also reported on laparoscopic surgery. Thereafter, many have reported on laparoscopic surgery for PEH. Although the high recurrence rate^2^ was initially a problem, there have been more and more reports on its feasibility and safety.^3^, ^4^

Most large hiatal hernias (LHH) are PEH. Once prolapse of the stomach to the chest cavity becomes manifested in PEH, it is called an intrathoracic stomach (ITS). Upside‐down stomach (UDS) is ITS with nearly 100% of the stomach prolapsed. In this article, we clarify the definition of LHH and ITS and provide a review on minimally invasive surgery for LHH from several viewpoints.

## 2 DEFINITION OF LHH

First, we clarify terminology such as PEH, LHH, giant hiatal hernia (GHH), ITS, UDS, and short esophagus (SE).

**Figure 1:**
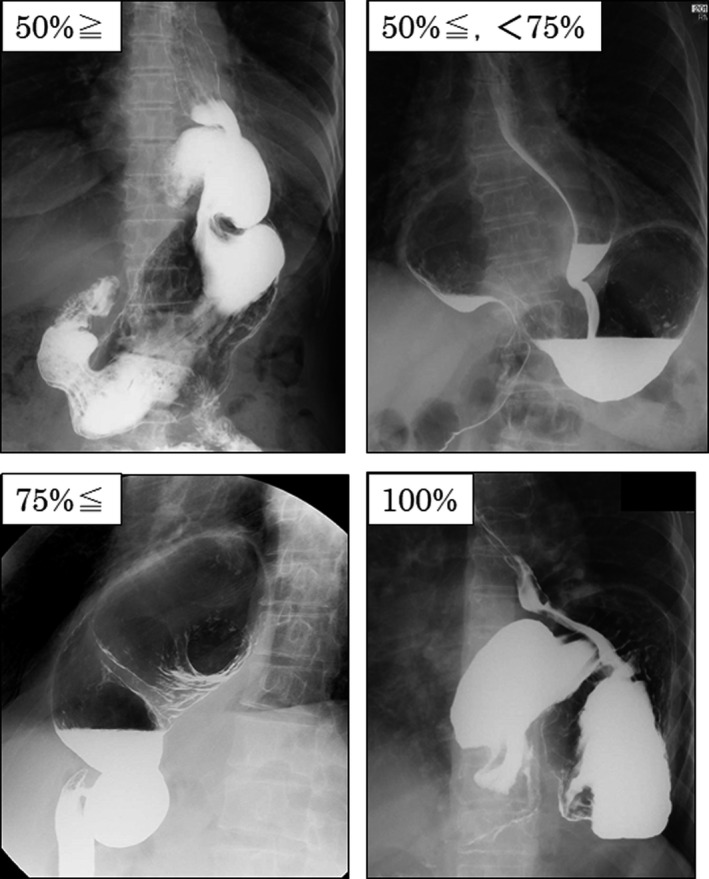
Paraesophageal hiatal hernia. Classification of paraesophageal hiatal hernia according to the degree of gastric prolapse to the chest cavity

There is no clear international definition for LHH. It is defined by articles in terms of the extent to which the stomach is prolapsed to the chest cavity (Figure 1). For example, one may define LHH as a stomach prolapse of 50% or more, which is categorized into 50% or more prolapse, 75% or more prolapse, and 100% prolapse. In this case, LHH is equivalent to PEH. On the other hand, SE is used when the esophagogastric junction is located 5 cm or more closer to the rostral side from the normal anatomical position^5^ and there are complications such as esophageal ulcer and stenosis as well as fibrosis of the tunica muscularis esophagi. Some argue that SE is carried out when sufficient length of the abdominal esophagus cannot be assured even if the esophagus in the mediastinal space is sufficiently detached during surgery.^6^, ^7^, ^8^ ITS means a prolapse of a substantial part of the stomach to the chest cavity, which is similar to LHH. GHH has almost the same meaning as LHH. Some assert that a prolapse of 50% or more of the stomach to the chest cavity is called GHH.^9^ Large PEH and giant PEH therefore mean a prolapse of 1/3 to 1/2 or more of the stomach to the chest cavity.^9^, ^10^, ^11^ UDS means prolapse of nearly the entire stomach. However, it is necessary to confirm the definition by articles because it does not necessarily indicate prolapse of the entire stomach.

**Figure 2:**
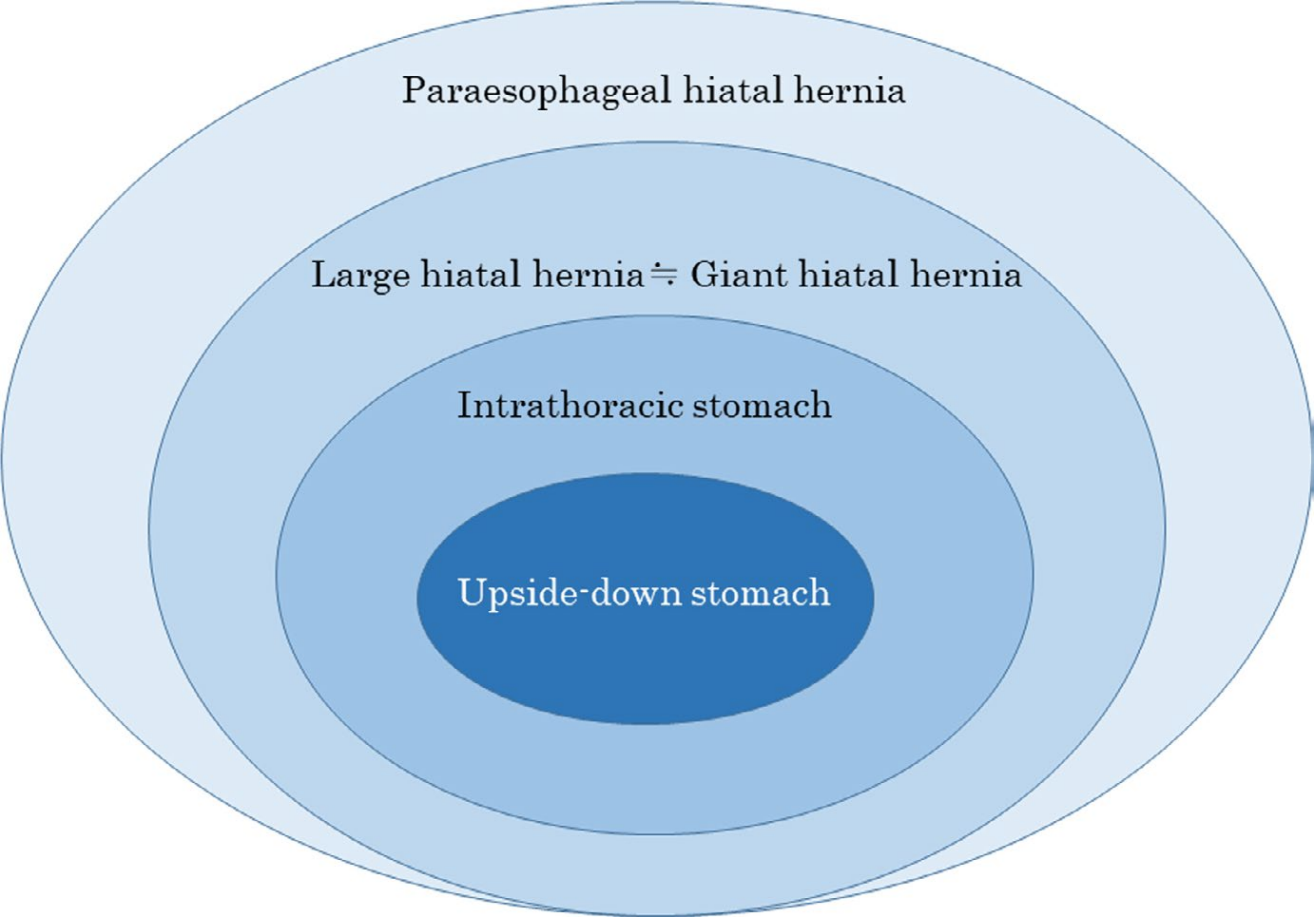
Concept of paraesophageal hiatal hernia. Regarding paraesophageal hiatal hernia, the extent of prolapse of the stomach to the chest cavity is as follows: upside‐down stomach ≥ intrathoracic stomach ≥ large hiatal hernia ≒ giant hiatal hernia

In summary, most analysis targets on LHH are PEH. Studies on LHH target subjects with a prolapse of approximately one‐third or more of the stomach to the chest cavity. Many studies on ITS target subjects with a prolapse of 50% to 75% or more of the stomach to the chest cavity.^12^, ^13^ Studies on UDS target subjects with a prolapse of 75% or more of the stomach to the chest cavity,^13^ some of which are limited to only 100%. Regarding PEH, the extent of prolapse of the stomach to the chest cavity is as follows: UDS ≥ ITS ≥ LHH ≒ GHH (Figure 2).

## 3 SURGICAL INDICATIONS FOR LHH

According to the guidelines by SAGES in 2013,^10^ “All symptomatic PEHs should be repaired, particularly those with acute obstructive symptoms or those that have undergone volvulus.” Many reports support this statement.^11^, ^14^ There is no disputing the surgical indication for symptoms of gastric outlet obstruction and torsion.

The statement “all symptomatic PEHs should be repaired” indicates the importance of the symptoms of PEH patients. The presence or absence of symptoms is controversial. There have been reports on relatively low incidence of symptoms such as heartburn 26%, postprandial chest discomfort or chest pain 23%, dysphagia 21%.^6^ Carrott et al divided 270 large‐PEH patients in accordance with the proportion of ITS into four groups: <50%; 50% or more; 75% or more; and 100%, then examined the symptoms in detail.^15^ The occurrence rates of the symptoms were as follows: heartburn: 76%‐56%; regurgitation: 54%‐38%; chest pain: 48%‐50%; and dysphagia: 42%‐54%. UDS comes with complications such as strangulation, stomach obstruction, acute bleeding from ulceration, leading to stomach necrosis, perforations and mediastinitis.^16^ Fifty percent or more of these patients have GERD symptoms.^17^ Going forward, further examination is necessary of the degree and frequency of symptoms.

Because LHH compresses the heart and lungs, it may cause a decline in cardiac function and respiratory function. However, it is difficult to conclude that LHH causes symptoms such as chest pain, palpitations, respiratory discomfort, and coughing. There have been reports on improved cardiac function by HH repair, along with reports on both‐side heart failure due to ITS. Carrott et al examined the respiratory function before and after surgery among 120 PEH patients, reporting that respiratory function was improved after surgery, such as forced vital capacity, forced expiratory volume in 1 second, vital capacity, and diffusion capacity of the lungs for carbon monoxide.^18^ This improvement was particularly manifested in patients aged 80 or older, patients with large hernias, and patients with decreased preoperative respiratory function. Therefore, disorders in cardiac function or respiratory function may be improved following surgery.

On the other hand, there is a statement which says, “routine elective repair of completely asymptomatic PEH may not always be indicated. Consideration for surgery should include the age and comorbidities of patients.” Some argue that we should consider the possibility of volvulus for ITS and UDS involving 75% or more stomach prolapse and carry out surgery even without symptoms.^19^ For ITS and UDS with a high degree of prolapse without any symptoms, we will consider age and quality of life (QOL) when it comes to surgery indication.

## 4 OUTCOMES OF SURGERY TREATMENT FOR LHH

We need to consider numerous factors when examining surgery treatment outcomes for LHH and PEH, so it is difficult to summarize the treatment outcomes.

The most important patient factors include the degree of stomach prolapse and the presence or absence of a SE. Many surgical factors are considered, including whether or not mesh should be used, the types of fundoplication and whether or not they should be used, and whether to use either emergency repair or elective repair.^11^ The evaluation of outcome also differs depending on articles in the definition of recurrence, whether it is recurrence of symptoms or anatomical recurrence. While the recurrence rate of hernias is relatively high following surgery for PEH,^20^, ^21^, ^22^, ^23^ the hernias are often mild, so many have reported them as not being a clinical problem.^24^ Consequently, the evaluation of outcomes is difficult. In this article, we evaluated the outcomes in terms of fundoplication, emergency versus elective, and mesh.

### 4.1 Fundoplication

The decision on whether or not fundoplication should be carried out following PEH repair, along with what procedures should be performed, is controversial. In general, fundoplication needs to be conducted to prevent postoperative GER, with intra‐abdominal gastric fixation recommended to reduce recurrence.^25^ On the other hand, some argue that the merits are unclear regarding conducting fundoplication on patients without reflux,^26^ while others have reported that fundoplication increased the postoperative incidence of dysphagia, which reached a maximum of 50% following PEH repair.^4^ Blake et al reported that fundoplication should not be carried out on patients without a history of significant reflux, or with poor esophageal motility, SE, or debilitating comorbidities.^13^ Others reported that the addition of fundoplication to ITS surgery does not contribute to QOL improvement,^27^ and that there was no difference in the symptom scores, satisfaction, and use of proton pump inhibitors in accordance with the presence or absence of anti‐reflux surgery (ARS).^6^ The guidelines state, “Fundoplication must be performed during repair of a sliding type hiatal hernia to address reflux. Fundoplication is also important during PEH repair.” It is believed that the addition of ARS to anatomical repair has greater merit. Heartburn, a typical symptom of GERD, was present in 26% to 76% of patients.^6^, ^15^, ^28^ Therefore, we believe ARS should be added for patients exhibiting GER symptoms prior to surgery.

Although the latest data show that 90% or more of the surgeries for PEH in the USA are laparoscopic surgery,^29^ whether or not laparoscopic surgery is optimal remains controversial. The Belsey Mark IV (BM‐IV) method, a transthoracic procedure, results in a high symptom disappearance rate.^12^ Laan et al^30^ conducted a retrospective study by matching 118 patients to the laparoscopic Nissen method and BM‐IV, respectively. Although the recurrence rate and surgery satisfaction were equivalent, the occurrence rate of esophageal leak and the rate of re‐surgery were higher in the Nissen group (6.8% vs 0%, 9.3% vs 2.5%, respectively). Laan et al stated that the BM‐IV method is more desirable for large PEH (Table 1).^30^ New facts may be found if thoracoscopic surgery is carried out going forward.

**Table 1:** Laparoscopic fundoplication and/or gastropexy for paraesophageal hiatal hernia

Author (Ref.)	Technique	Patients	Age (y)	Hernia type	Mesh	Operative time (min)	Length of stay (d)	Morbidity (%)	Mortality (%)	Follow‐up (mo)	Post‐op esophagitis	Hernia recurrence (%)	Satisfaction (%)	Re‐operation (%)
Park et al^32^	Hill	29	55.2	PEH	No	—	—	—	—	147	—	—	85	10.3
Ponsky et al^33^	AG with Toupet (27)/Dor (1)	28	67	III	No	146	2 (1‐14)	11	0	21 (3‐24)	—	0	100	—
Muller‐Stitch et al^38^	MAH with C	20	65	III	Yes	124	7.8 (4‐25)	15	0	12	53	33	100	—
	MAH with F	20	63	III	Yes	153	8 (4‐21)	5	0	12	17	17	94	—
Laan et al^30^	BM‐IV	118	71	ITS ≥ 75%	No	202.5	6 (4‐42)	21.2	0	22.2	—	8.4	83	2.5
	Nissen	118	71.9	ITS ≥ 75%:44.9% ITS ≥ 50%:55.1%	No	202	3 (1‐68)	22	0.9	32	—	16.1	72	9.3
Huerta et al^36^	Nissen	117	64	III/IV = 78/22	Almost yes	175	2 (1‐3)	23	—	58.5	—	4	72	—
	Toupet	62	65	III/IV = 84/16	Almost yes	166	2 (1‐2.5)	26	—	25	—	3	67	0

Abbreviations: AG, anterior gastropexy; BM‐IV, Belsey Mark IV (not laparoscopic approach); C, cardiophrenicopexy; F, fundoplication; ITS, intrathoracic stomach; MAH, mesh‐augmented hiatoplasty; PEH, paraesophageal hiatal hernia.

The fixation method of the stomach, either by posterior fixation or anterior fixation, is also open to question. Hill proposed the Hill method from the idea that maintaining cardiophrenic angles is essential for the control of GER.^31^ Park et al compared the cases that underwent laparoscopic Hill surgery for PEH and could be followed up for a long term with cases that underwent laparoscopic Hill surgery for GERD during the same period, reporting there was no difference in the symptoms between the two groups (Table 1).^32^ Although there was no mention of the recurrence rate, the satisfaction rate was high at 85%. While the Hill method involves posterior fixation, there have also been reports of anterior fixation. Ponsky et al reported that they carried out fundoplication and anterior fixation by suturing and fixing two sites on the stomach anterior wall and abdominal wall. The result was that all cases became free of symptoms and had no recurrence.^33^

Broeders et al^34^ compared the Nissen method and anterior fundoplication. Meta‐analysis of five randomized controlled trials (RCTs) revealed that although two groups had equivalent anti‐reflux effects, the anterior fundoplication indicated less severity of dysphagia following surgery, suggesting the significance of anterior fundoplication. Tian et al compared the Nissen method and Toupet method in 13 RCTs, demonstrating the significance of the Toupet method.^35^ Although the subjects of these studies were patients with GERD and the results cannot be completely applied to PEH patients, as long as there are no differences in the anti‐reflux effects depending on the type of fundoplication, it is desirable to choose the procedure resulting in less dysphagia following surgery. Huerta et al compared the long‐term outcome of the Nissen method and the Toupet method among 77 PEH patients, reporting that there was no difference in symptom‐reducing action and surgery satisfaction.^36^ According to Furnée et al, not performing fundoplication resulted in the occurrence of abnormal esophagus acid reflux in 39.3% of patients following surgery and the occurrence of reflux esophagitis in 28% of patients.^37^ Müller‐Stich et al compared the groups undergoing hiatus reinforcement using mesh with cardiophrenicopexy alone and the group undergoing reinforcement with fundoplication in an RCT. The postoperative incidence of esophagitis was 53.3% versus 16.7%, indicating the significance of fundoplication.^38^ Several reports have recommended anterior hemifundoplication for UDS.^17^, ^39^ For UDS, Vrba et al carried out Nissen fundoplication on patients with preoperative reflux symptoms or reflux esophagitis while conducting fundopexy on the remaining cases, which resulted in good outcomes.^16^

Based on the above, keeping in mind that fundoplication should be carried out in general, decisions should be made by comprehensively taking into consideration the esophageal motility of each patent, the severity of GERD, age, and complications.

### 4.2 Mesh reinforcement

The usefulness of mesh is controversial. There are two statements on it in the guidelines. One is, “The use of mesh for reinforcement of large hiatal hernia repairs leads to decreased short‐term recurrence rates.” Although mesh involves complications, it reduces short‐term recurrence rates.^8^, ^40^ The other statement is, “There are inadequate long‐term data on which to base a recommendation either for or against the use of mesh at the hiatus.” Recently, a report using the data from a period of 48 months following surgery indicated that the use of mesh decreased the recurrence rate.

Mesh may involve serious complications.^17^, ^41^, ^42^ Typical complications include esophageal erosion and penetration, along with increased risk of infections.^18^ Fatal complications reportedly include strangulation of re‐herniated stomachs through a narrowed hiatus reconstructed with mesh and aortal bleeding. Surprisingly, prosthetic mesh reinforcement was reported to require re‐surgery for 23 of 28 patients and esophagectomy for seven patients.^41^ It was pointed out that the reason for this high rate of complications included two aspects: the procedure of mesh indwelling; and the material of the mesh. Moreover, when the opening of the hiatus esophagus is large and crural repair cannot be performed, using mesh to bridge the hiatal defect may lead to serious complications.^42^ In addition, the suture and tacking during mesh fixation may involve serious complications such as cardiac tamponade. Recently, there have been fewer reports on serious complication compared to the past.^40^, ^43^, ^44^ It has also been reported that not using prosthesis mesh resulted in a low recurrence rate and good treatment outcomes.^11^

During the early 2000s, there were several RCTs but no reports on mesh‐related complications.^21^, ^45^ All of these reported that the use of mesh resulted in a decreased recurrence rate (26% vs 8%, 22% vs 0%).^21^, ^45^ Thereafter, while many have reported on the usefulness of mesh,^20^, ^44^ the long‐term outcome remains unclear.

Recently, there have been several reports on the meta‐analysis of mesh. Antoniou et al^8^ compared suture repair and biologic mesh repair among 295 subjects in six articles. The results revealed that the short‐term recurrence rate was 16.6% versus 3.5% (odds ratio [OR] 3.74, 95% CI 1.55‐8.98, *P* = .003). Although this indicates the short‐term usefulness of biologic mesh, the data were insufficient to indicate the long‐term outcomes. According to Tam et al,^46^ the postoperative recurrence of suture cruroplasty (SC) was 24% (91/382), while that of mesh cruroplasty was 13% (46/354). Although the rate of re‐surgery was 6% versus 3.7%, this rate was significantly higher in SC among cases in which the postoperative course could be evaluated (73% vs 53%). They concluded that quality of evidence supporting the routine use of mesh cruroplasty is low and the use of mesh should be left to the discretion of the surgeon until the evaluation of symptomatic outcomes and long‐term recurrence is clarified. Memon et al^43^ compared 186 SC subjects and 220 subjects with prosthetic hiatal herniorrhaphy (PHH) based on four RCTs. As a result, while the rate of re‐surgery was lower in the PHH group (OR 3.73, 95% CI 1.18, 11.82, *P* = .03), there was no difference in the recurrence of hiatal hernia or wrap migration (OR 2.01, 95% CI 0.92, 4.39, *P* = .07). The outcomes of the two procedures for LHH were almost equivalent. Memon et al were opposed to routinely conducting PHH.

The long‐term usefulness of mesh remains open to discussion 5 years or more after the publication of the guidelines in 2013. First and foremost, we should increase the rate of follow‐up. It is also desirable to clarify the definition of recurrence across institutions and conduct studies in institutions having abundant experience.

### 4.3 Emergency surgery versus elective surgery

Paraesophageal hiatal hernias involve complications such as gastric perforations, bleeding, and necrosis due to torsion.^47^ The occurrence rate of complications is relatively high without treatment.^6^, ^48^

Using The New York Statewide Planning and Research Cooperative System administrative database, Polomsky et al conducted an analysis among 4858 PEH patients:^49^ of females and 53% of patients were emergency visits, and 66% of the patients were discharged prior to surgery. The emergency patients included a high percentage of elderly individuals and a higher mortality rate than elective admission patients (2.7% vs 1.2%). They had a longer duration of admission (7.3 days vs 4.9 days) and the treatment costs thereof were high. The operative mortality rate was higher in emergency patients (5.1% vs 1.1%). Multivariate statistical analysis showed that the independent prognosis factors of death during hospitalization were age, emergency visit, and surgery. Moreover, Bhayani et al extracted 2756 subjects who underwent PEH repair from the National Surgical Quality Improvement Program data base (2005‐2010) and conducted an analysis of 412 patients with obstructed PEH (15%), comparing the treatment outcomes in the group who underwent early surgery within 1 day (57%) and the group who underwent late surgery in 1 day or later (43%).^47^ As patient characteristics, the proportion of patients with American Society of Anesthesiologists ≥ 3 was higher in the late surgery group. While the death rate had no significant difference (3.4% vs 4.6%), morbidity was significantly higher in the late surgery group (17% vs 30%): pneumonia (5.1% vs 9.7%), reintubation (2.1% vs 6.3%), and deep venous thrombosis (2.1% vs 6.3%).

In the study on ITS patients by Polomsky et al,^50^ the subjects included 104 patients who underwent elective repair and 23 patients who underwent emergent repair. The mortality rate was 22% versus 1%. They pointed out that the mortality and morbidity rates of emergent repair for ITS were higher than that of elective repair. This article reviewed the treatment for ITS from 1995 to 2009. The ratio of emergent repair was 20% (2.1%‐100%) on average. While the mortality of elective repair was 0.2% (0%‐1.0%), that of emergent repair was high at 6.5% (0%‐21.7%). Wirsching et al^51^ conducted a study among 570 PEH patients. Among 38 patients making emergency visits (6.7%), only three patients underwent emergency surgery, while the remaining were able to undergo semi‐elective surgery by conducting decompression of the stomach internal pressure. This article reviewed nine articles from 2008 to 2016, among which eight reported that emergency surgery increased morbidity or mortality, indicating that emergency surgery should be avoided as much as possible. On the other hand, few reports argue that the outcomes are good regardless of whether they are emergencies or semi‐urgent.^52^

The majority of reports recommend elective surgery.^13.28^ Those operating should be skilled and experienced surgeons.^15^ Surgeries in experienced centers reportedly resulted in low morbidity.

## 5 RELAXING INCISION

LHH often involve an open and large hiatus esophagus. Particularly for PEH, hiatus reefing often involves excessive tension. Mesh reinforcement after reefing with too much tension results in a high recurrence rate of hernia.^53^ Therefore, we perform treatment to relax this tension by making a relaxing incision for these cases.^54^, ^55^, ^56^, ^57^, ^58^, ^59^ However, there have been few reports on relaxing incisions so evaluations on the incision site and the effects thereof are insufficient.

The criteria for making a relaxing incision have not been established as yet. It is used when bringing the left and right crus closer during surgery and there is too much tension thereon. In Western countries, it is common to make a relaxing incision between the right crus and the inferior vena cava, a reportedly simple procedure.^53^, ^54^, ^55^, ^56^, ^58^ When the site at which a relaxing incision is to be made has inadequate intensity, is scarred, or the distance between the inferior vena cava and the right crus is close, the relaxing incision should be made on the left side of the hiatus esophagus, but it may be placed on either side when the lateral incision cannot result in adequate relaxation.^53^, ^54^, ^55^, ^56^, ^58^ On the other hand, Yano et al made the incision on the left side to avoid injuring the inferior vena cava.^57^ For making a relaxing incision on the right side, attention should be paid to avoid injuring the anterior crural vein or the thoracic duct. Make the incision along the right crus toward the right chest cavity. Regarding the left side, avoiding injuring the left phrenic nerves is essential. While Yano et al made an incision along the left crus, approximately 1 to 2 cm from the lateral margin of the left crus, it is common in Western countries to make an incision between the left crus and the seventh rib, sometimes reaching the lateral side beyond the spleen to obtain sufficient relaxing effects.^53^, ^57^, ^58^ After the incision, hiatal reinforcement using mesh is added.

Table 2 shows the surgical outcomes in accordance with the sites of the relaxing incision. Among all 46 cases, a right incision was made in 33 cases (72%), a left incision was made in 10 cases (22%), and an incision was made on both sides in three cases (6%). Biologic mesh was used in 36 cases (78%), while synthetic mesh was used in 10 cases (22%). There were no reports on procedural accidents during surgery, suggesting no problems with the safety of surgery.

**Table 2:** Surgical outcomes by the location of the relaxation incision

Author	Samples	Location	Mesh	Perioperative complications	Median follow‐up mo (range)	Recurrence	Re‐revision
Crespin et al^54^	16	Right: 12	Biologic	None	9 (6‐83)	6 (50%)	0
		Left: 3				2 (67%)	2 (67%)
		Bilateral:1				1 (100%)	0
Greene et al^55^	15	Right: 13	Biologic:10 Synthetic:5	None	15 (1‐27)	1 (7%, location is unknown)	0
		Left: 1					
		Bilateral:1					
Alicuben et al^56^	10	Right: 8	Biologic	N/A	5 (unknown)	1 (13%)	0
		Left: 1				0	
		Bilateral:1				0	
Yano et al^57^	5	Left: 5	Synthetic	None	13 (9‐24)	2 (40%)	0

Abbreviation: NA, not available.

Thirteen cases (28%) experienced recurrence during the postoperative follow‐up period. Among the 12 cases excluding one with an unknown site of recurrence, the recurrence was on the right side in seven cases (58%), the left side in four cases (33%), and both sides in one case (9%). The recurrence rate was 21% on the right side (7/33), 40% on the left side (4/10), and 33% on the both sides (1/3). The two cases requiring re‐surgery (4%) due to a diaphragmatic hernia were carried out on the left side.^54^ Among 31 cases excluding the reports in which the details on the mesh used were unknown,^55^ there was no significant difference in the recurrence rate between the mesh types (biologic mesh 38% [10/26], synthetic mesh 40% [2/5]). However, Crespin et al^54^ recommend using synthetic mesh for the prevention of postoperative diaphragmatic hernias when making a relaxing incision on the left side.

The decreased rate of tension after making a relaxing incision was reported to be 46% on one side and 56% on both sides.^58^, ^59^ A relaxing incision enables crural repair in a tension‐free state, leading to an anticipated decrease in the recurrence rate. However, the usefulness of making a relaxing incision remains unproven for the prevention of LHH recurrence. The recurrence rates after primary closure, primary closure + biologic mesh and relaxing incision + biologic mesh were 58%, 38% and 56%.^54^

From the above, while making a relaxing incision is useful for primary tension‐free closure during surgery when there is too much tension during crural repair, it has yet to contribute to improvement in the recurrence rate.

## 6 COLLIS GASTROPLASTY

SE arises from chronic acid reflux leading to fibrillation and constriction of the esophagus.^6^ While the incidence of SE was reportedly 1.53% among GERD patients and 11.87% among PEH patients,^25^ it has also been reported that severe transmural esophagitis and fibrosis resulting in true esophageal shortening is a very rare consequence.^17^ According to the guidelines, “At the completion of hiatal repair, the intra‐abdominal esophagus should measure at least 2‐3 cm in length to decrease the chance of recurrence. This length can be achieved by mediastinal dissection of the esophagus and/or gastroplasty.” It is essential to ensure the length of intra‐abdominal esophagus for carrying out ARS.

In the event that SE was diagnosed after sac excision and sufficient mediastinal dissection of the esophagus during operation for ITS, as Blake et al^13^ suggests, Collis gastroplasty (CG) and fundoplication should be carried out if the esophageal motility is ensured. Nason et al^7^ also carried out CG if they were unable to ensure that the intra‐abdominal esophagus was tension‐free. Many argue that CG should be performed when an intra‐abdominal esophagus cannot be adequately ensured. On the other hand, it has been reported that CG is not necessary by conducting mediastinal esophageal mobilization,^60^ and that Hill gastropexy combined with Nissen fundoplication resulted in an outcome equivalent to Collis‐Nissen (CN).^61^

In the surgery for SE, it is necessary to create a neoesophagus as an intra‐abdominal esophagus. For the creation thereof, a laparoscope can be used on its own or in combination. When a laparoscope is used on its own, the methods include the wedge technique^62^, ^63^ proposed by Terry^64^ and Whitson,^65^ a method involving creating small stomata on the upper body of the stomach body using an end‐to‐end anastomosis stapler and dissecting the stomach from the stomata toward the esophageal longitudinal axis,^66^ and a method involving approaching the laparoscope and dissecting the stomach along the esophageal longitudinal axis.^67^ Regarding fundoplication, floppy Nissen and Toupet fundoplication are selected based on the esophageal motility evaluation.^7^

CN surgery as a form of minimally invasive surgery was reported in 1996 to have good outcomes in three cases.^5^ Mattioli et al reviewed the outcomes of minimally invasive CN in 2015.^67^ According to the authors, as the deviation of the number of cases was large (3‐454; median 15), they extracted five reports including 50 cases or more.^62^, ^63^ These reports showed good outcomes as follows: mortality 0%‐1.7%; morbidity 8%‐28%; postoperative dysphagia 0%‐37%; postoperative reflux symptoms 1.9%‐28%; and anatomical recurrence 2.5%‐16.6%, with a satisfaction level of 93%‐98%.

From the above, the CG is effective when it is difficult to ensure an intra‐abdominal esophagus.

This article summarized the current state of ARS, as it may be carried out going forward for large PEH, CG, and redo ARS, in which it is difficult to carry out laparoscopic surgery.

## 7 CONCLUSION

The feasibility and safety of laparoscopic repair for LHH and PEH have been nearly established. Although the outcomes of elective repair are obviously better than emergency repair, attention should be paid to performing elective repair on patients with no symptoms, taking into consideration their age and complications. Although not a major clinical problem, following PEH repair, the rate of anatomical recurrence increases with age. Although it has been a long time since the clinical application of mesh, the long‐term effectiveness of mesh remains controversial. We anticipate an early conclusion to this problem by increasing the rate of follow‐up as much as possible.

## DISCLOSURE

Conflicts of Interest: Authors declare no conflicts of interest for this article.

Author Contributions: NO wrote the manuscript and made tables and figures. KT and FY supervised the section of “fundoplication” and “relaxing incision”, respectively.
